# On the effect of adding clinical samples to validation studies of patient-reported outcome item banks: a simulation study

**DOI:** 10.1007/s11136-015-1199-9

**Published:** 2015-12-08

**Authors:** Niels Smits

**Affiliations:** Research Institute of Child Development and Education, University of Amsterdam, Nieuwe Achtergracht 127, 1018 WS Amsterdam, The Netherlands

**Keywords:** Item response theory, PROMIS, Item banks, Quasi-traits, Sampling

## Abstract

**Purpose:**

To increase the precision of estimated item parameters of item response theory models for patient-reported outcomes, general population samples are often enriched with samples of clinical respondents. Calibration studies provide little information on how this sampling scheme is incorporated into model estimation. In a small simulation study the impact of ignoring the oversampling of clinical respondents on item and person parameters is illustrated.

**Method:**

Simulations were performed using two scenarios. Under the first it was assumed that regular and clinical respondents form two distinct distributions; under the second it was assumed that they form a single distribution. A synthetic item bank with quasi-trait characteristics was created, and item scores were generated from this bank for samples with varying percentages of clinical respondents. Proper (using a multi-group model, and sample weights, respectively, for Scenarios 1 and 2) and improper (ignoring oversampling) approaches for dealing with the clinical sample were contrasted using correlations and differences between true and estimated parameters.

**Results:**

Under the first scenario, ignoring the sampling scheme resulted in overestimation of both item and person parameters with bias decreasing with higher percentages of clinical respondents. Under the second, location and person parameters were underestimated with bias increasing in size with increasing percentage of clinical respondents. Under both scenarios, the standard error of the latent trait estimate was generally underestimated.

**Conclusion:**

Ignoring the addition of extra clinical respondents leads to bias in item and person parameters, which may lead to biased norms and unreliable CAT scores. An appeal is made for researchers to provide more information on how clinical samples are incorporated in model estimation.

## Introduction

In the last decade computerized adaptive testing (CAT) has become a popular method for efficiently assessing patient-reported outcomes (PROs). For example, in the USA, the patient-reported outcomes measurement information system (PROMIS), a group of scientist from several academic institutions and the National Institutes of Health [1], has developed a multitude of CATs for the measurement of health status for physical, mental, and social well-being for use in clinical research and healthcare delivery settings [2]. In Europe, similar initiatives have been taken (e.g., [3]) which have resulted in CATs for measuring depression and anxiety [4–6]. Moreover, the substantive number of presentations on CAT at the most recent meeting of the International Society for Quality of Life Research [7] shows that many CATs for measuring PROs are currently being constructed, and it may thus be concluded that it has become a part of both clinical practice and research.

At the core of CAT lies item response theory (IRT), a group of models in which answers to questionnaire items are modeled by specifying separate parameters for representing item properties and patient characteristics. The patient characteristic is commonly referred to as “latent trait,” and denoted by parameter *θ*. The items usually have two types of parameters: parameter *a* represents the extent to which the item is able to discriminate between different levels of the latent trait; parameter *b* is a location parameter which is expressed on the same scale as the latent trait and indicates the position on which a higher response category is preferred over a lower response category. The latent trait scale is arbitrary by definition [8], and a linear transformation of a chosen scale results in identical expected item category probabilities. Commonly, IRT software anchors the scale by putting it on a *z*-score scale, setting the mean and standard deviation of the latent trait to zero and one, respectively [9, Chap. 6].

CATs commonly utilize item pools that are calibrated using a random sample from the target population (e.g., [2]). If the latent trait is bell-shaped in this population, the *z*-score scale may be directly used to create norms [10], and CAT results may be presented as a percentile rank or *T*-score [11]. However, PROs are often quasi-traits [12, 13], which means that items are not informative on the low end of the scale. When using a general population sample for calibration, this results in highly skewed item scores (i.e., few people endorsing items at moderate to high levels of severity) by which the measurement properties at the right-hand side of the latent trait continuum are imprecisely captured. A solution that is most often taken is to enrich the calibration sample with samples from clinical populations (i.e., patients known to score high on the latent trait) (e.g., [14–17]). Adding extra respondents seems harmless because it is often stated that the item parameters of IRT models are population independent (e.g., [9, Chap. 2]), but that is not true for the models commonly estimated for PROs [18]. Therefore, to provide sound general population norms, this sampling scheme should be properly incorporated in the calibration. Unfortunately, in the experience of the author, calibration studies provide little detail on how this matter is dealt with, which makes it hard to evaluate if CAT results conform to valid population norms.

The purpose of this study is to show how population norms may be biased if the inclusion of clinical samples in calibration studies is improperly dealt with (i.e., if the oversampling is not incorporated in the model). To that end, a small simulation study using synthetic data is presented which shows the impact on latent trait estimates as a function of the percentage of clinical respondents in the calibration sample. In standard IRT modeling two approaches may be taken to incorporate the data of the clinical sample: First, the general population and clinical samples are treated as two distinct distributions, and second, a single distribution is assumed for both groups, and the clinical sample consists of persons who score higher on the latent continuum. In the simulation study data were generated under both scenarios and the impact of improperly dealing with the inclusion of extra clinical samples was investigated. Which approach should be chosen for a specific item bank is not the topic of this study; such a choice should be made on the basis of substantive and/or empirical grounds.

## Method

### Item bank properties

Throughout the simulation a single synthetic item bank for generating item scores was used. For its construction the setup of [19] was adopted, which was based on a thorough survey of calibration studies for quasi-traits. Here, the most important characteristics are described; for a detailed description the reader is referred to [19]. The item bank consisted of forty 5-point Likert scale items complying with the graded response model (GRM, [20]), a model for polytomous items. GRM models the score on a *K*-category item via *K* − 1 cumulative probabilities. The *k*th cumulative probability specifies the probability that category *k* + 1 or higher is chosen. The GRM for a person with latent trait *θ* and five-category item is of the form:

1 $$P_{k}(\theta )=\frac{{\rm e}^{a(\theta -b_{k})}}{1+{\rm e}^{a(\theta -b_{k})}},\quad k=1,2,3,4,$$

where *a* is the item discrimination parameter and the set of location parameters $b_k$ gives the boundaries on the latent trait scale above which one is expected to prefer a higher over a lower category.

A summary of the distribution of the item bank’s true GRM parameter values is given in Table 1, which confirms its quasi-trait status: Because location parameters were mostly placed at the upper side of the latent trait scale, the bank provided little information on the lower side of the scale.

**Table 1:** Distribution of GRM item parameters in the simulated 40-item bank

	*a*	*b*_1_	*b*_2_	*b*_3_	*b*_4_
Min	1.04	−0.97	0.27	1.20	2.20
Mean	1.89	−0.11	0.90	1.93	2.94
Max	2.66	0.89	1.52	2.72	3.57

### Generating calibration samples

To obtain responses from the item bank, the true item parameters and *θ*s were entered in Eq. (1), giving four cumulative probabilities for each item. Next, these were transformed into item category probabilities, and a category was randomly drawn from the resulting multinomial distribution.

Each of the generated calibration samples consisted of 1500 simulated persons (i.e., 1500 *θ* values were drawn; details are provided below). This sample size was chosen because previous research has shown that sample sizes of at least 1000–2000 provide precise estimates of GRM item parameters [21].

In addition, to investigate the impact of the relative size of clinical respondents in the calibration sample, three percentages of clinical respondents were used: 25, 50 and 75 %, respectively. The first two values were in the neighborhood of the percentages used in several PROMIS calibrations (e.g., 37 % in [14, 16] and 40 % in [15, 17]). The third value, 75 %, may be encountered in situations in which data are mainly obtained at clinical institutions (e.g., [5, 22]).

Several perspectives on the nature of the latent variables underlying PROs exist (e.g., [23, 24]), but there are two that dominate the literature, which will be used in the simulation. First, respondents from general and clinical populations make up different categories, and second, they originate from a single distribution.

#### Scenario 1: Two distinct distributions

**Fig. 1:**
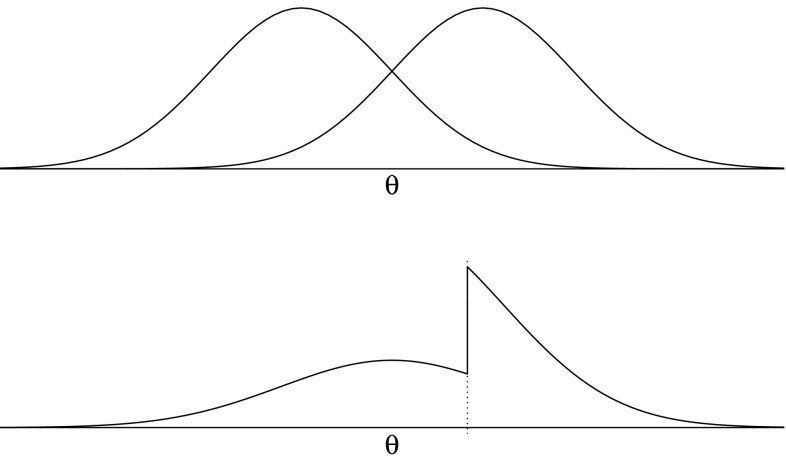
Distributions for generating *θ* under the two scenarios. In the *upper panel* (Scenario 1) two distributions are assumed: a general population (*left*) and clinical population (*right*). In the *lower panel* (Scenario 2) a single distribution is assumed; the clinical region of the scale is at the *right-hand side* of a critical value and clearly has an inflated density

Under the first scenario, the general and clinical populations have distinct but overlapping distributions of the latent trait (also see [25]). For some PROs, calibration studies have suggested that this assumption is legitimate (e.g., [15, 26–28]). The upper panel of Fig. 1 shows the distributions from which *θ*s were sampled. In the simulation, the general population had a normal distribution with a mean of −2 and SD of 1; the clinical population had a normal distribution with a mean of 0 and SD of 1. The percentage of clinical respondents was controlled by varying the number of draws from both distributions; for example, in the 50 % condition, 750 *θ*s were sampled from each population.

#### Scenario 2: One distribution

Under the second scenario, the latent trait scores of clinical and general populations can be described by a single distribution; respondents scoring above a critical value suffer from pathology and thus belong to the clinical group (e.g., [29]). The critical value depends upon prevalence, i.e., the proportion of the population found to have pathology. Under this scenario, the adding of extra clinical respondents to the calibration sample would implicitly change the shape of the distribution from which it is sampled. The lower panel of Fig. 1 shows the distribution in the case of half of the sample consisting of clinical respondents. In the simulation, the standard normal distribution (i.e., a mean of 0 and standard deviation of 1) was used to sample from. The critical value was set at 1.28, which corresponds with a prevalence of pathology of 10 % in the general population (in the simulation, this critical value is assumed to be known). Regular respondents were sampled from the resulting left-hand region, and clinical respondents were sampled from the right-hand region. The percentage of clinical respondents was controlled by varying the number of draws from both regions; for example, in the 50 % condition, 750 *θ*s were sampled from each region. It should be noted that prevalence should be distinguished from the rate of clinical respondents in the calibration sample, as the former rate is associated with a population and the latter with a sample. One of the goals of the current simulation is to show how estimates may be biased if the two rates are unequal due to the sampling scheme.

### Calibration approaches

For each scenario, the proper way of dealing with the clinical sample was compared with a default approach which ignored the sampling scheme. Under this approach, GRM was run for the entire matrix of simulated item response data in each calibration set, i.e., for all 1500 simulees and 40 items. Location and discrimination parameter estimates were obtained using marginal maximum likelihood [30], which assumes that the latent trait follows a (single) standard normal distribution.

Under the first scenario, the proper way of dealing with the clinical sample would be to fit a multi-group model (e.g., [31, Chap. 15]). In the simulation, a multi-group GRM was estimated assuming two normal distributions of *θ*, one for the general population and one for the clinical population. To allow for a metric comparable to that of the true item parameters, the mean of the clinical population was fixed at zero, and the general population value was freely estimated; SDs of the latent trait in both populations were assumed to be equal to 1.

Under the second scenario, the proper way of dealing with the additional clinical sample would be to use sampling weights (e.g., [32]). It is clear from Fig. 1 that the distribution has too much mass at the right-hand side of the critical value, and observations from this region should be down-weighted to obtain the original normal distribution. To do so, the IRT model must give a small weight to the item scores of respondents who are oversampled and a large weight to the item scores of respondents who are undersampled (e.g., [33]). For example, in the 50 % clinical respondents condition, 750 respondents were sampled from both the regular and the clinical regions. By contrast, prevalence is 10 % (i.e., the target population consists for 10 % of clinical subjects and for 90 % of regular subjects), and therefore, under random sampling the expected numbers of observations would be 150 and 1350, respectively. Under the 50–50 sampling scheme, the probability of being sampled for clinical respondents is therefore 5 (50/10) times as high as under random sampling (under which it should be equal to prevalence), and for regular respondents it is 0.56 (50/90) times as high. The proper weights to be used in the IRT model are the inverses of these probability ratios: Clinical respondents get a weight of 0.2 (10/50) and regular respondents get a weight of 1.8 (90/50). Note that the sum of weights is equal to the total sample size.

Under each of the two scenarios, two types of calibrations (a proper and an improper way of dealing with the clinical sample) were performed for each percentage of clinical respondents (25, 50 and 75 %), resulting in a total of 12 sets of item parameter estimates. Models were estimated using the mirt library [34, 35] in R [36].

### Outcome variables

Two outcomes were studied: recovery of item parameters and recovery of person parameters. To study the recovery of item parameters, the estimates resulting from each calibration were contrasted with the true (i.e., generating) parameters. To that end, two measures were used: the correlation between the true parameter values and their estimates, and the mean difference between the true parameter values and their estimates.

To study the recovery of person parameters, new data were generated from the original item bank (i.e., using the true item parameters). Five hundred subjects were generated at each of 17 evenly spaced true *θ*-values from −4 to 4 with an increment of 0.50, and item responses were created as described above. Next, these responses were used to estimate *θ*s under each of the twelve calibrations. Estimation was performed using maximum a posteriori (MAP, [9]), which uses a standard normal prior distribution. This Bayesian method is popular in PRO measurement because it allows for estimates for perfect not-endorsed response patterns, which are often encountered when measuring quasi-traits (by contrast, maximum likelihood estimation would be problematic because under that method finite estimates for these patterns cannot be obtained). Two measures were used to study recovery of person parameters: the correlation between the true parameter values and their estimates, and the mean difference between the two. In addition, the latter outcome was studied as a function of the true estimate. Finally, because the standard error of the estimated *θ* is often used as a stopping criterion in CATs, the estimated standard error was studied as well.

## Results

### Scenario 1: Two distinct distributions

Table 2 shows the item parameter recovery results under the first scenario. The left-hand (right-hand) side of the table shows the outcomes under the incorrect (correct) approach of dealing with the clinical sample. Both approaches showed high correlations between the true and estimated parameters, and values were very similar between the two approaches. Correlations were highest for the *b*_1_ and lowest for the *b*_4_ parameters. Under the correct approach (the two-group model), the estimates were close to the true parameters (i.e., the mean differences between true and estimated values were close to zero). By contrast, under the incorrect approach (the one-group model), the parameter estimates were clearly biased (nearly all mean differences deviated from zero substantially). All *a* parameters were overestimated (the mean difference between true and estimated *a*s was negative), whereas the pattern for the location parameters varied for the different levels of percentage of clinical respondents. For example, for *b*_4_ the difference was negative in the 25 % clinical respondents condition and positive in the other two conditions. Clearly, under the improper calibration approach the scaling of the latent trait was different from the true scaling. The incorrect assumption of a single distribution yields a scale with an origin which lies in between the means of the two true distributions and a unit which is about equal to the overall standard deviation of the combined populations.^1^

**Table 2:** Item parameter recovery under Scenario 1 (two distributions)

Parameter	One-group model		Two-group model	
	Cor (true, estimate)	Mean (true–estimate)	Cor (true, estimate)	Mean (true–estimate)
*Percentage of clinical respondents is 25 %*				
*a*	0.967	−0.772	0.966	0.021
*b*_1_	0.990	−1.105	0.990	0.039
*b*_2_	0.986	−0.801	0.986	0.045
*b*_3_	0.935	−0.516	0.937	0.016
*b*_4_	0.833	−0.224	0.834	0.006
*Percentage of clinical respondents is 50 %*				
*a*	0.983	−0.759	0.983	0.040
*b*_1_	0.993	−0.751	0.993	−0.032
*b*_2_	0.988	−0.474	0.988	−0.069
*b*_3_	0.972	−0.189	0.972	−0.101
*b*_4_	0.926	0.078	0.926	−0.150
*Percentage of clinical respondents is 75 %*				
*a*	0.974	−0.564	0.975	0.027
*b*_1_	0.996	−0.382	0.996	0.037
*b*_2_	0.994	−0.164	0.994	0.008
*b*_3_	0.974	0.058	0.973	−0.024
*b*_4_	0.922	0.271	0.922	−0.061

Cor (*a*, *b*) is the correlation between *a* and *b*

**Fig. 2:**
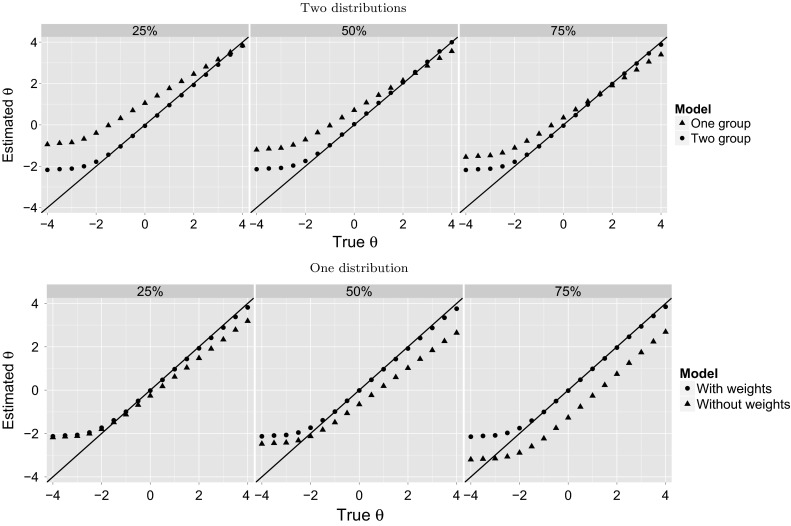
Relation between true and estimated *θ* under Scenario 1 (*upper panel*) and Scenario 2 (*lower panel*) for the three percentages of clinical respondents. The *straight line* is the line of equality ($y=x$)

The upper panel of Table 3 presents the person parameter recovery results. Under both approaches the correlation between true and estimated latent trait values was high, although under the one-group model, the correlations were marginally higher than under the two-group model. Under both models the estimates of the latent trait were biased, although the average deviance was substantially larger under the one-group model. The upper panel of Fig. 2 shows mean estimated *θ* as a function of true *θ*. Under the one-group (i.e., incorrect) model approach the latent trait was overestimated substantially for most parts of the scale, except for the upper extreme region, where some underestimation was found; the size of the bias decreased with increasing levels of percentage of clinical respondents. In addition, to provide some meaning to the size of bias, let us inspect the estimates at a true *θ* of 2, as it is the analog of a *T*-score of 70 (corresponding to a percentile rank of 98), a common critical value in clinical assessment [37]. In the 25 and 50 % conditions the mean estimate of *θ* was obviously too high (comparable to *T*-scores of about 75 and 72, respectively), and severity was therefore overestimated, whereas in the 75 % condition bias was almost absent.

**Table 3:** Person parameter recovery under Scenario 1 (upper panel) and Scenario 2 (lower panel)

Clinical percentage (%)	One-group model			Two-group model		
	Cor ($\theta ,\hat{\theta }$)	Mean ($\theta -\hat{\theta }$)	Mean (SE)	Cor ($\theta ,\hat{\theta }$)	Mean ($\theta -\hat{\theta }$)	Mean (SE)
*Two distributions*						
25	0.988	−1.137	0.237	0.981	−0.238	0.257
50	0.987	−0.828	0.227	0.981	−0.324	0.260
75	0.985	−0.516	0.227	0.981	−0.259	0.258

Clinical percentage (%)	Model without weights			Model with weights		
	Cor ($\theta ,\hat{\theta }$)	Mean ($\theta -\hat{\theta }$)	Mean (SE)	Cor ($\theta ,\hat{\theta }$)	Mean ($\theta -\hat{\theta }$)	Mean (SE)
*One distribution*						
25	0.982	0.017	0.233	0.981	−0.261	0.255
50	0.982	0.417	0.222	0.982	−0.254	0.255
75	0.977	0.911	0.242	0.981	−0.268	0.258

Cor (*a*, *b*) is the correlation between *a* and *b*

Under the two-group (i.e., correct) model, the estimated values were unbiased for *θ*s ranging from −1.5 to 4.0, but for *θ*s below −1.5 estimates were too high, and bias increased with decreasing *θ*. The bias on the lower side of the scale resulted from the item bank having little information for this region. As a result much weight was given to the prior distribution employed by MAP, which pulled the estimates toward zero (a phenomenon commonly known as ‘shrinkage’).

Examining the standard errors of the estimated *θ*s in Table 3, the one-group model gave lower mean values than the two-group model, which suggests that when CATs are constructed under the improper approach, measurement precision is overestimated and therefore adaptive testing stops too early.

### Scenario 2: One distribution

Table 4 shows the item parameter recovery results for the second scenario. The left-hand (right-hand) side of the table shows the outcomes under the incorrect (correct) approach of dealing with the clinical sample. Both approaches showed high correlations between the true and estimated item parameters, and values were very similar between the two approaches although most of the correlations were marginally higher for the model without weights. Again, correlations were smallest for the *b*_4_ parameter. Under the proper approach (i.e., using weights) the item parameters were unbiased, having mean differences of about zero. By contrast, under the improper approach biased estimates were found. The *a* parameter was clearly overestimated in the 25 and 50 % condition, whereas it was slightly underestimated in the 75 % condition; the location parameters were underestimated in all three conditions, with bias increasing with increasing levels of percentage of clinical respondents. The incorrect assumption of random sampling yields a scale with an origin which lies at the right-hand side of the true mean and a unit which is about equal to the standard deviation of the unweighted true distribution.

**Table 4:** Item parameter recovery under Scenario 2 (one distribution)

Parameter	Model without weights		Model with weights	
	Cor (true, estimate)	Mean (true–estimate)	Cor (true, estimate)	Mean (true–estimate)
*Percentage of clinical respondents is 25 %*				
*a*	0.980	−0.281	0.978	−0.024
*b*_1_	0.995	0.242	0.995	0.009
*b*_2_	0.994	0.372	0.993	0.020
*b*_3_	0.979	0.494	0.973	0.022
*b*_4_	0.953	0.586	0.938	0.005
*Percentage of clinical respondents is 50 %*				
*a*	0.986	−0.342	0.980	−0.029
*b*_1_	0.991	0.640	0.990	0.003
*b*_2_	0.996	0.806	0.991	0.027
*b*_3_	0.993	0.965	0.981	0.046
*b*_4_	0.973	1.097	0.946	0.030
*Percentage of clinical respondents is 75 %*				
*a*	0.981	0.062	0.960	0.011
*b*_1_	0.993	1.289	0.989	0.015
*b*_2_	0.994	1.289	0.981	−0.007
*b*_3_	0.994	1.248	0.946	−0.007
*b*_4_	0.968	1.207	0.885	−0.049

Cor (*a*, *b*) is the correlation between *a* and *b*

The lower panel of Table 3 presents the person parameter recovery results. Under both approaches the correlation between true and estimated latent trait values was high, and between the approaches very similar. Under both approaches the estimates of the latent trait were biased, although the average difference varied with percentage of clinical respondents for the models without weights but not for the models with weights. The lower panel of Fig. 2 shows mean estimated *θ* as a function of true *θ*.

Under the model with weights (i.e., proper approach), patterns were highly similar to the two-group model of the first scenario in that the latent trait was unbiased except for the lower left part (*θ* < −1.5). Under the model without weights (i.e., improper approach) the latent trait was mostly underestimated, except for the lower extreme region, where it was overestimated; bias increased with higher rates of clinical respondents, with substantial values in the 50 and 75 % conditions. In addition, to give more meaning to the size of bias, let us again inspect the estimates at a true *θ* of 2 (comparable to a *T*-score of 70). In all three conditions the mean estimate of *θ* was obviously too low (comparable to *T*-scores of 65, 60 and 57, respectively), and severity was therefore underestimated.

Turning to the standard errors of the estimated *θ*s in Table 3, the model without weights gave lower mean values than the model with weights; again, this suggests that when CATs are constructed under the improper approach, measurement precision is overestimated and therefore adaptive testing stops too early.

## Discussion

In this simulation study, the effect of improperly dealing with the addition of extra clinical samples in the validation of PRO item banks was studied. Simulations were performed under two scenarios: First, clinical and general population respondents constitute two distinct populations; second, they jointly make up a single distribution. In addition, under both scenarios the percentage of clinical respondents in the calibration sample was varied. Although under the improper approaches the correlation between true and estimated parameters was generally very high, these estimates were clearly biased. Under the first scenario, when the models were estimated assuming one instead of two groups, the discrimination and location parameters were overestimated. Likewise, person parameters were overestimated, with bias decreasing in size with increasing percentage of clinical respondents. Under the second scenario, when the models were estimated without the required weights, the discrimination parameters were mostly overestimated and the location and person parameters were underestimated with bias increasing in size with increasing percentage of clinical respondents in the calibration sample. In addition, the standard error of the estimated latent trait value was generally underestimated under both scenarios.

It should be noted that the goal of the current study was not to provide an exact quantification of the size of the potential bias in latent trait estimates in calibration studies using additional clinical samples, but to give insight into how bias may emerge when the sampling scheme is ignored. The outcomes showed that the size of bias was a function of scenario (one versus two assumed distributions), rate of clinical respondents in the calibration sample, and the true value of *θ*. Under the first scenario, ignoring the sampling scheme generally led to *over*estimation, whereas under the second scenario it leads to *under*estimation. In the first scenario, bias was smallest when the rate of clinical respondents in the calibration sample was *high*, whereas in the second scenario, bias was minimal when this rate was *low*. Likewise, under the first scenario bias was most substantial at the low end of *θ*, whereas under the second scenario bias was most substantial at the high end of the scale. To make the size of the bias more interpretable, *θ* may be transformed to a *T*-score scale and evaluated at a commonly used critical value of 70; the highest mean estimate in the first scenario corresponded to a *T*-score of 75, and in the second scenario the lowest mean value corresponded to a *T*-score of 57.

This study showed that ignoring the sampling scheme may lead to structural over- or underestimation of item parameters and therefore to biased latent trait estimates. As a consequence, the norm-scores used in clinical practice, such as percentile ranks and *T*-scores, may be too high or too low, which may lead to over- or under-diagnosing of complaints. In addition, it also showed that scores may not only be biased but may also be less reliable if adaptive testing is used. As the standard errors calculated under the improper calibrations were generally too low, and the size of the standard error is commonly employed as stopping criterion in CAT, stopping may occur too early resulting in scores that are less reliable than required.

The simulation study suggested the following effects of item parameter estimates on person parameters and their precision. Bias in location parameters resulted in a similar bias (under- or overestimation) in the latent trait estimate. In addition, overestimation of discrimination parameters was associated with underestimation of the standard error of the latent trait. As the amount of test information is known to increase with higher values of the *a* parameter (see, [38]), it is to be expected that the standard error decreases because it is inversely related to test information.

Even when the sampling scheme was properly dealt with, the latent trait was generally overestimated for *θ* < −1.5. This is a result of the combination of an item bank measuring a quasi-trait and the Bayesian nature of MAP estimation. By assuming a prior distribution, the estimates were pulled toward the center of the population distribution [39], zero in this case. This issue is consequential for some PRO assessment goals, such as the measurement of change: changes from or toward the left side of the scale may remain unnoticed. It may therefore be concluded that although Bayesian methods solve one problem (i.e., they provide finite estimates for perfect response patterns), they introduce another problem (i.e., bias) [40]. It may thus be fruitful to consider other methods to deal with perfect response patterns, such as restricting the range of values for the estimated latent trait so that maximum likelihood estimation provides finite estimates (for example, setting a lower bound for $\hat{\theta }$ of −4.0) (e.g., [41]).

In the current study, data were generated and evaluated under two popular perspectives on the nature of the latent trait. Although both views allowed for generating item responses which resembled those typically found for quasi-traits, this pair of perspectives is not comprehensive. For example, under both views normal distributions were assumed, whereas recent research has suggested that some latent traits underlying PROs may be skewed [18, 42] or unipolar [43] which would call for additional simulation perspectives. It may be argued, however, that under alternative perspectives, ignoring the addition of extra clinical respondents would lead to biased norms as well and that the main message from this study would therefore not change by adding perspectives. Similarly, several choices were made for other factors of the simulation design, such as the percentage of clinical respondents in the calibration sample and the size of prevalence in the second scenario, which may not match all calibration situations. Although other choices would have given different numbers, it may be stated that the general pattern of results would not have been very different. For example, another simulation using a prevalence of 25 % instead of 10 % (results not shown herein) yielded very similar outcomes.

This paper shows the importance of properly dealing with extra clinical samples and it is recommended that future calibration studies provide more information on the approach taken for dealing with the issue so that its appropriateness may be evaluated.

## Footnotes

1 Under both scenarios the estimated location parameter values can be roughly predicted through a linear transformation: estimated parameter $=\bar{\theta }^*+\sigma ^*\,\times$ true parameter, where $\bar{\theta }^*$ and $\sigma ^*$ are the overall mean and overall standard deviation of the population distribution, respectively.
